# Evolution at the Origins of Life?

**DOI:** 10.3390/life14020175

**Published:** 2024-01-24

**Authors:** Ludo L. J. Schoenmakers, Thomas A. C. Reydon, Andreas Kirschning

**Affiliations:** 1 Konrad Lorenz Institute for Evolution and Cognition Research (KLI), 3400 Klosterneuburg, Austria; 2 Institute of Philosophy, Centre for Ethics and Law in the Life Sciences (CELLS), Leibniz University Hannover, 30159 Hannover, Germany; thomas.reydon@cells.uni-hannover.de; 3 Institute of Organic Chemistry, Leibniz University Hannover, 30167 Hannover, Germany; andreas.kirschning@oci.uni-hannover.de

**Keywords:** origins of life, evolutionary theory, generalization, reduction, chemical evolution, metabolic networks, protocells

## Abstract

The role of evolutionary theory at the origin of life is an extensively debated topic. The origin and early development of life is usually separated into a prebiotic phase and a protocellular phase, ultimately leading to the Last Universal Common Ancestor. Most likely, the Last Universal Common Ancestor was subject to Darwinian evolution, but the question remains to what extent Darwinian evolution applies to the prebiotic and protocellular phases. In this review, we reflect on the current status of evolutionary theory in origins of life research by bringing together philosophy of science, evolutionary biology, and empirical research in the origins field. We explore the various ways in which evolutionary theory has been extended beyond biology; we look at how these extensions apply to the prebiotic development of (proto)metabolism; and we investigate how the terminology from evolutionary theory is currently being employed in state-of-the-art origins of life research. In doing so, we identify some of the current obstacles to an evolutionary account of the origins of life, as well as open up new avenues of research.

## 1. Introduction

The theory of evolution is one of the most successful modern scientific theories. It allows us to explain why there is such a great variety of living beings, why living beings tend to be functionally well-organized and adapted to their environments, how living beings are related historically, and, perhaps most importantly, it provides a deep understanding of the natural processes that have gone into creating the diversity that we find in the biological world. Evolutionary theory is also a uniquely multifaceted scientific theory, combining insights from many different fields over its now more than 150-year history. Charles Darwin’s version of the theory, often referred to as *classical Darwinism* in his honor, drew from disciplines such as geology, paleontology, and zoology [1]. During the early 20th century, classical Darwinism was gradually extended into what would ultimately come to be known as the *Modern Synthesis* [2], which combined Darwin’s original theory with Mendelian and population genetics. During the latter half of the 20th century, the increasing use of chemistry and physics in biology extended our understanding of evolution to the molecular level, which was seen by many as confirmation that the Modern Synthesis was essentially on the right track. At the same time, a number of further conceptual innovations, as well as the combination of evolutionary biology with insights from developmental biology (EvoDevo) and ecology (EcoEvoDevo), resulted in efforts to modify the theory of evolution, known under the umbrella name of the *Extended Evolutionary Synthesis* [3,4,5]. The Extended Synthesis is meant to be an improvement upon the Modern Synthesis in the sense of being a richer theory that acknowledges a broader variety of explanatory factors.

Given the measure of its success, the broadness of both its explanatory and disciplinary scope, and the relative simplicity of the central Darwinian logic, it is not unsurprising that there have been many attempts to extend evolutionary thinking beyond the realm of biology [6]. Evolutionary research programs have been launched in economics, literary studies, psychology, computing science, and many other disciplines [7,8]. Many of these research programs stand at a great ontological distance from the kind of organismal biology on which evolutionary theory was originally based. However, evolutionary theory, its language, and its concepts, are also increasingly applied to fields that are more closely related to organismal biology, particularly in origins of life research, synthetic biology, and minimal biology [9,10,11].

This raises several interesting questions: How, if at all, can evolutionary theory be fruitfully extended to the origins of life? Did living systems become evolutionary when crossing some important threshold sometime after life originated, or were living systems evolutionary from the start? Provided that life started with prebiotic chemistry and transitioned through a protocellular phase, to what extent has each of these phases been evolutionary? In other words, did evolution start before, at, or after the origin of life? How, if at all, is the current use of evolutionary terminology in origins of life research justified? And finally, what do answers to these questions tell us about the possibilities for extending or generalizing evolutionary theory beyond biology more generally?

In this review, we explore these empirical, theoretical, and philosophical questions in an effort to begin elucidating the role of evolution at the origins of life. Our aim is to set an agenda for further research, so rather than providing detailed answers to each of the above questions, we highlight central issues and avenues for further work. In Section 2, we discuss some of the most influential applications of evolutionary theory beyond organismal biology to date, and we identify some of the pitfalls that these efforts face. In Section 3, we review certain chemical aspects of the origins of life, particularly in relation to the role of catalysis in (proto)metabolism. In Section 4, we discuss the application of evolutionary language and concepts in current empirical research into the origins of life.

Before we begin, four disclaimers are in order. First, in this review, we make use of both philosophical and scientific insights to tease out what we think is relevant to the application of evolutionary theory to the origins of life. Given the breadth of this topic, no review of this kind is going to be complete and in the present paper we will discuss those issues that we believe to be central. Second, philosophical reviews tend to have a more opining character than scientific reviews, as empirical results on their own are less likely to lead to a philosophical position being either rejected or justified. This review is no exception. Third, most of the views that we discuss here have only one thing in common: they are not widely accepted. Elements of particular theories that are discussed in each section might be generally accepted, but no particular theory presented here finds near universal or even majority assent. We take this as an important indicator that the application of evolutionary theory to the origins of life is very much a field that is still in flux and in which no dominant paradigm has been established yet. Fourth, it is important to note that our concern is with the applicability (or lack thereof) of evolutionary theory to prebiotic chemistry at the origin of life. Our aim is to provide an overview of the various ways in which evolutionary theory has been suggested to apply at and before the origin of life and, in particular, to highlight difficulties in such applications that call for further conceptual, theoretical, and empirical work. Our concern, thus, is emphatically not with the origins of the genetic code, which is a much more specific question than the one we are addressing here. The genetic code is the chemical key to living systems and constitutes the chemical basis of many evolutionary processes, and various hypotheses are available in the literature regarding its origins and its relation to the Tree of Life, which have been reviewed elsewhere [12,13,14,15]. One approach is focused on the role of symmetry in the standard genetic code and beyond [16]. The standard genetic code consists of 61 codons, arranged as triplets of the four bases (A, U, G, and C for mRNA), which encode for 20 amino acids. This means that most amino acids are encoded by two or more codons, often differing in the third-position base (a concept known as degeneracy). Recent work has been focused on understanding the various symmetries that can be found in the genetic code, in relation to the physicochemical properties of the amino acids [17], DNA quadruplet symmetry [18], and using Fibonacci-like sequences to derive various chemical properties of the code and the associated amino acids [19].

However, tracing the origin of the genetic code is a much more specific and very different question from asking whether the prebiotic chemistry that eventually led to the origin of biological evolutionary processes can also be understood as constituting genuinely evolutionary processes. The research that we discuss in what follows is concerned with (possibly evolutionary) chemical processes that well preceded the origin of the genetic code and, even though relevant for the question of the genetic code, deals with aspects of prebiotic chemistry that are not or only indirectly connected to questions regarding the genetic code.

## 2. Evolutionary Theory beyond Biology

### 2.1. Some Central Questions

Modern evolutionary theory is a grand and multifaceted theory. Since the publication of Darwin’s *Origin of Species*, the theory has continuously been developed and expanded upon, and the next major revision is currently being debated [3,4]. Current evolutionary biology encompasses a considerable variety of ways of describing evolutionary processes that involve different central concepts and focus on different explanatory factors. Rather than applying the full breadth of evolutionary concepts, principles, methodologies, and disciplinary insights, however, applications of evolutionary theory beyond biology are often based on some version of Evolution by Natural Selection (ENS). One of the earliest and most famous formulations of ENS is due to Lewontin [20] (p. 1): “Darwin’s scheme embodies three principles: 1. Different individuals in a population have different morphologies, physiologies, and behaviors (phenotypic variation). 2. Different phenotypes have different rates of survival and reproduction in different environments (differential fitness). 3. There is a correlation between parents and offspring in the contribution of each to future generations (fitness is heritable)”. Whether this precise formulation is justified or not—and various alternative formulations have been proposed [21]—it is meant to express that evolution requires a minimal set of three ingredients: variation of characteristics, relevance of those characteristics to fitness, and heritability of those characteristics. ENS is often seen as the core of the Darwinian logic, and it is this austere version of evolutionary theory that is often taken as the starting point by those applying evolutionary theory outside of biology. Indeed, Lewontin suggested that ENS is a generally applicable principle, since it does not presuppose any specific material substrate or any specific mechanism in which the principle is implemented.

This immediately raises two important, related questions, even before we look at actual applications of ENS in non-biological or near-biological fields (i.e., fields dealing with aspects of the living world while not being a part of the biological sciences strictly speaking). First, is ENS on its own sufficient to capture the large variety of paradigmatic examples of biological populations undergoing evolution? Godfrey-Smith [22] has provided reasons why ENS on its own is too minimal to adequately cover the relevant range of evolving biological populations. Briefly, Godfrey-Smith argues that biological populations, and possibly non-biological populations too, can be more or less Darwinian (i.e., can exhibit Darwinian evolution to various degrees) based on a number of different but concrete parameters that are not captured completely in “classical” ENS accounts. Godfrey-Smith’s arguments amount to the observation that biological evolution manifests itself in a multitude of ways in a large variety of populations, such that adequately describing evolutionary processes requires more than just the principle of ENS.

Second, assuming that some version of ENS indeed captures at least a large part of the relevant paradigmatic cases, is natural selection all that is required for us to apply the predicate “evolutionary” to the relevant processes in some non- or near-biological field? This question is closely connected to one of the most hotly debated topics in evolutionary theory, namely the relative importance of ENS compared to other evolutionary forces such as drift or developmental constraints. *Adaptationism* is the view that natural selection is the primary and perhaps exclusive causal force in evolution, i.e., natural selection ultimately determines which traits organisms have [23]. Adaptationism can be further distinguished into at least three kinds [24], also showing the three ways in which we can be anti-adaptationists: (1) empirical adaptationism, which is the empirical claim that natural selection is in fact the most important or only causal factor in evolution; (2) explanatory adaptationism, which is the view that ENS is the answer to *the* central explanandum in evolutionary biology, namely the apparent design of organisms; and (3) methodological adaptationism, which is the view that, whatever else determines the design of organisms, we ought to start by looking at traits as adaptations as explained by ENS, and only invoke other explanatory factors if explanations invoking natural selection are insufficiently supported. We can see immediately that how we think about these three versions of adaptationism determines in part whether and how we accept ENS as the central part of evolutionary theory that could in principle be extended to a different domain. 

Attempts at extending evolutionary theory beyond biology usually do not explicitly take the above considerations into account, hence our brief discussion. Yet we take it as self-evident that the application of evolutionary theory beyond biology—in the form of ENS or otherwise—must depart from a defensible biological understanding of evolution. The biological world is the origin of evolutionary theory and any subsequent extension to another domain must be consistent with evolutionary theory as understood in biology in order for it to be evolutionary at all (in the technical sense of the term), although this consistency may come in different forms. Some authors object to a requirement that applications outside biology must be consistent with how evolutionary theory is used in biology and argue that biological evolutionary theory itself is merely a special case of an overarching theory that is not specifically biological [25]. But this does not resolve the issue. Those who argue that evolutionary theory itself is captured in a yet more fundamental or overarching theory face the challenge of showing how evolutionary theory as applied to biology, in its full empirical complexity, flows out of this more fundamental or overarching theory.

In the next sections, we discuss several examples of the application of evolutionary theory outside biology—both in the “upwards” direction of the social realm, as well as the “downwards” direction of the molecular origins of life. Our reason for doing so is that both directions provide interesting clues about what is required for evolutionary theory to make sense when applied outside biology proper. We separate these accounts into two groups, which is not intended as an exhaustive classification of accounts but rather as an organizational principle. The first group consists of *generalizing accounts*. These accounts are characterized by the extension of the central Darwinian logic—usually understood as ENS—from organismal biology to whichever other field, process, or set of phenomena seems to fit that logic. Thus, Darwinian evolution is not restricted to the biological world, but ranges over a larger portion of reality. Approaches that fall into this group should in principle be applicable in the context of origins of life research, as they are intended to apply to all systems that exhibit evolutionary behavior. However, generalizing accounts tend not to be applied to possible cases of evolution at the origin of life. This may be a missed opportunity.

The second group consists of *reducing accounts*, which come in two varieties. Contrary to generalizing accounts, *strong reducing accounts* of evolutionary theory take the central Darwinian logic itself to be reducible to some further set of chemical and/or physical principles. In typical reductive fashion, these are then taken to explain evolutionary phenomena from the smallest physicochemical components, or most fundamental physicochemical principles, upwards. *Weak reducing accounts* are reducing in the sense that the emphasis lies on a physicochemical understanding of biological life and its adaptive diversity, but these accounts differ from strong reducing accounts in that evolutionary theory at the biological level is not reduced to physics or chemistry, even though the physical and chemical level is causally important to how evolution at the biological level is to be understood.

### 2.2. Generalizing Accounts

Generalizing accounts tend to be broad sweeping and far-reaching, which is particularly true once evolutionary theory is applied to higher-order behavioral phenomena such as language, social structures, or culture. Two prominent examples of this sort of account are the evolutionary research programs of *Organizational Ecology* [26,27] and *Generalized Darwinism* [28,29].

Organizational Ecology is a research program in organizational studies that is aimed at providing an ecology-inspired analysis of populations of organizations (i.e., firms, NGOs, and other institutions) [26,27]. The central question in this program is how the variety of organizational forms that we observe in social reality are best explained. This question parallels the question regarding organismal variety in biology, such that it seems that an evolutionary approach can be applied fruitfully here. How the “ecology-inspired” aspect of the program should be understood precisely has not always been made clear by those involved in the program, but a general interpretation of Organizational Ecology is that it is an *evolutionary* program that centers on ENS and understands organizations as products of an evolutionary process that adapted them to their economic and social environments [30]. On this view, the variety of organizational forms is best explained by selection processes on organizational populations, that is, processes that are governed by the principle of ENS or at least processes that are sufficiently similar. Although this similarity between biological and organizational evolution should not be overstated (cf. [31]), it seems clear that Organizational Ecology does at least entail a minimal extension of evolutionary theory beyond organismal biology. 

Criticism of Organization Ecology has been focused on one of the ontological requirements for this program to count as “evolutionary”. Reydon and Scholz [30] have argued that organizational populations are not populations of the right sort to be subject to evolution. Crucially, organizational populations are conceived of as groups or sets of similar entities, whereas biological populations are determined by reproductive interactions between their member organisms and by common descent. No comparable interactions exist between the member organizations of organizational populations. Thus, there is an ontological mismatch between evolutionary biology and Organizational Ecology. As evolutionary biology is the paradigm case, this means that Organizational Ecology cannot be considered evolutionary in the technical sense of the term.

Generalized Darwinism [28,29] is a paradigmatic example of a generalizing account. It was developed within the context of evolutionary economics but aims to be applicable more broadly. It takes the same evolutionary processes that occur in the biological realm to occur in the social realm, making many social processes of change essentially Darwinian, meaning along ENS lines. Social populations, at whatever level they occur, are subject to evolution if variation in heritable traits of their members (people, social institutions and organizations, and so on) confers fitness differences. This approach has been criticized on similar grounds as Organizational Ecology: it remains unclear *how* the members of social populations reproduce, how they interact in ways that are similar to the interactions between members of biological populations, and how they can be said to share a common descent in the same robust way that biological populations do [32]. Moreover, Generalized Darwinism has been criticized for failing to meet two requirements for the fruitful explanatory transfer of evolutionary theory to the social realm [7]. Briefly, these requirements are: (1) sufficient similarity between that which is to be explained, i.e., phenomena in the social realm must be sufficiently similar to those in the biological realm (similarity of explanandum criterion); (2) sufficient similarity between that which does the explaining, i.e., how supposedly evolutionary processes are explained in the social realm must be sufficiently similar to how evolutionary processes are actually explained in biology (similarity of explanans criterion).

Organizational Ecology and Generalized Darwinism are two examples of generalizing theories that might be said to go upwards in complexity, extending the application of evolutionary theory to entities at levels of organization that are above the organism level (in this case covering complex social phenomena). As such, they encounter a specific set of questions that pertain to, among other things, the existence of populations at higher levels of organization and the occurrence of relations of reproduction and descent between entities at these levels. Other generalizing accounts go both upwards and downwards in complexity, also covering the early stages of life. Here, Dennett’s [33] notion of Darwinism as a *Universal Acid*, Dawkins’ [34] and Hull’s [35] *Replicator View*, and Maynard-Smith and Szathmary’s [36,37] notion of *Major Evolutionary Transitions* (METs) are important examples. 

Dennett views the central logic of Darwinism as the single most important discovery in human intellectual history [33]. Natural selection, Dennett points out, is a fully general algorithmic process capable of producing apparent design of ever-increasing complexity. Moreover, it is substrate neutral, meaning that any entity that fits the logic of natural selection—which in Dennett’s view is just about everything, in one way or another—is subject to evolution involving the principle of ENS. Dennett’s algorithmic view of Darwinian evolution involves an understanding of ENS in terms of replicators, after an idea that was originally developed by Dawkins [34] and later Hull [35]. On this view, a replicator is any entity which can make copies of itself. DNA molecules are paradigmatic examples, but there is nothing that prevents other entities at various levels of organization from being replicators in this sense. Self-replicating entities can be selected based on their replicative success in their specific environment, which is usually understood as how quickly and accurately a replicator reproduces, how many copies of itself it produces, and how stable the replicator itself is (how long the replicator and its copy replicators tend to endure). At some point in evolution, replicator cooperation becomes advantageous in comparison to individual replication and collections of replicators build “vehicles” (in Dawkins’ terminology, “interactors” in Hull’s terminology) to further their replicative success. On the Replicator View, the first primordial self-replicators (whatever molecules these may have been) eventually developed into RNA and DNA molecules, which are the crucial, self-replicating entities that are subject to selection in the biological evolutionary process, and the builders of organisms that serve as their vehicles. The algorithmic process that started at a molecular level thus slowly extended to higher levels of organization. Importantly, given substrate neutrality, the notion of selection of replicators can also be extended to the level of cultural evolution as the currently highest level at which evolution is thought to occur. Here, the selection, spread, and change of ideas, or “memes”, through large human populations (and possibly the entire world population) and the origin of cultural traits, as well as social institutions and structures, are taken to be subject to the same Darwinian logic that applies at the molecular and organismal levels.

The Replicator View is also prominent in Maynard-Smith and Szathmary’s [36,37] account of Major Evolutionary Transitions. The MET account is an account of several complex phenomena, ranging from the origins of the first cells to the origins of language. Important in this account is the role of *unlimited hereditary replicators*, which are understood as polymeric coding entities that allow for an unlimited number of molecular phrases to be produced. Here, Maynard-Smith and Szathmary view RNA as the victorious unlimited hereditary replicator, although other hereditary replicators might have preceded it. These replicators are crucial for ENS—which the authors view as the core of evolutionary theory. However, while the transition of prebiotic chemistry to replicator chemistry might be described as “chemical evolution”, on the MET account, it would not be seen as evolution proper, precisely because the prebiotic phase of the origins of life lacked unlimited hereditary replicators.

The replicator-based ENS accounts presented above are attractive from an origins of life perspective, but they are not without their problems. From the perspective of evolutionary biology, such views have been criticized as being overly adaptationist [38,39]. Natural selection is an important factor in evolution, perhaps even the most important factor in explaining adaptive change in populations, but it is not the only important factor, and the gene may not be the unit of selection of choice. Bringing us closer to the origins of life, for example, Godfrey-Smith [22] has argued that replicators are not essential for ENS, because replicators require perfect inheritance, and perfect inheritance itself is not a prerequisite for ENS. In response, Bourrat [40] has argued that perfect inheritance is essential in *purely* adaptive examples of evolution, where ENS is the *only* relevant factor, and has argued that the Replicator View corresponds to such a purely adaptive perspective. This perspective, which is an idealization of the evolutionary process that reduces evolution to natural selection, might be more relevant at the origins of life than at later stages of the evolution of life on Earth. Finally, there is the empirical challenge of actually showing that a robustly self-replicating molecule can be constructed (RNA-based or otherwise) and that this molecule can indeed lead to increasingly complex systems through ENS. This challenge is partially due to the question of how accurate replication must be for evolutionary processes to occur. While it is clear that molecules exist that can be copied in chemical processes, the question here is how accurate such copying processes must be to be able to speak of *self*-replication. Copying in DNA replication, for instance, is not perfect and imperfect copying gives rise to the variation that drives evolution. Still, we think of genes as being passed on to subsequent generations—which we would not do if copying were imperfect to an extent that copies had entirely different sequences from the original. What is required, thus, is a general account of what self-replication consists in that accommodates the degrees of imperfect copying that occur in actual evolutionary processes.

### 2.3. Reducing Accounts

On a strong reducing account, evolutionary theory can be reduced to some more fundamental chemical or physical theory, which then explains the Darwinian nature of reality from the ground upwards. Two examples of this approach are the *Dynamic Kinetic Stability* (DKS) approach [41], which draws heavily on reaction kinetics, and what we may call the *Formal Reduction* approach [42,43,44], which draws heavily on thermodynamics and physical theories of learning. We discuss these two accounts in Section 2.3.1. In Section 2.3.2, we briefly consider two weak reducing accounts, namely Eigen and Schuster’s *Hypercycle* approach [45,46] and Kauffman’s *Complex Self-Organization* view [47].

#### 2.3.1. Strong Reducing Accounts

On the DKS approach, originally developed by Pross and Khodorkovsky [48], evolutionary theory at the biological level is “just the biological manifestation of a broader physicochemical description of natural forces.” [21] (p. xiii). Pross’ favored physicochemical description is that of reaction kinetics in combination with thermodynamics. While Pross develops this account across a large number of publications, the central notion of dynamic kinetic stability is most cogently defended in [49]. Pross views the fact that living systems are in thermodynamic states far from equilibrium as the most important difference between living and non-living matter. This state runs counter to what would be expected based on classical thermodynamics, namely that the sum total of all reactions in any living system moves towards equilibrium, which is the lowest free energy state of the system and therefore thermodynamically the most stable state in which the system persists. How do living systems manage to exist in states that are removed from the equilibrium state? According to Pross, they do so by attaining a different kind of stability—where Pross understands stability broadly as a lack of change through time—namely dynamic kinetic stability through replication. Because of the ongoing turnover of members in populations of replicators (dynamic), the replicator system as a whole attains a kind of stability of form, one that is based on the continuous extraction of free energy from its environment, as governed by kinetic parameters. At the organizational level of the system (the population of replicators), a stable state of ongoing turnover can thus be achieved, which is not at the lowest free energy state. According to Pross, this abstract physicochemical description generally holds for the behavior of systems (populations) consisting of replicators. Therefore, it can in principle be generalized to encompass all of biology, effectively reducing biology to chemistry [50]. Pross admits that quantification of the DKS account (and with that the actual generalization of the account for all replicator systems) remains challenging, such that the possibility of an actual reduction of biology to chemistry remains an open question. 

The formal reduction approach of Vanchurin and coauthors [42,43,44] seeks to present a formal, general theory of evolution, using thermodynamics and what they consider to be a physical theory of learning as its starting points. Vanchurin and coauthors here use a very general concept of learning as the accumulation by a system of information about its environment (as used in the area of machine learning), which they see as a general characteristic of evolving systems. Interestingly, the formal reduction approach is structured similarly to the DKS approach. It focuses on the fact that from the perspective of thermodynamics, living systems are expected to go towards thermodynamic equilibrium, but this is not the case, at least locally. Therefore, there must be a force driving them away from their equilibrium state. In the formal reduction approach, the increase in entropy due to the second law of thermodynamics is offset by a decrease in entropy in evolving systems due to what the authors call the “second law of learning” [43]. Living systems are considered to be learning entities, that is, information-accumulating entities that are governed by the relevant physics (in particular thermodynamics and information theory), such that the applicable laws of physics can provide a physical understanding of biological evolution. Unlike the DKS approach, the authors argue that their theory can be used to make quantifiable predictions and is therefore empirically testable [43]. Similar to the DKS approach, the formal reduction approach is exceedingly generalist and reductionist, with the authors going as far as stating that their theory “can potentially apply to the entire history of the evolving universe” wherein life is but “a specific, even if highly remarkable form.” [24] (p. 2). 

Pross’ DKS approach and the formal reductionism of Vanchurin and coauthors currently stand as some of the most extensively developed reducing accounts of early evolution, particularly in relation to the origins of life. However, in part due to their specialized nature, it is challenging to assess these accounts on their evolutionary merits. This is shown by the fact that, unlike the generalizing but non-reductionist approaches of Organizational Ecology and Generalized Darwinism, the Darwinian pretensions of these approaches have not yet received critical attention. Moreover, it has yet to be shown *how* precisely biology reduces to chemistry or physics in these two approaches, and what that means for biological (evolutionary) research. To note just two reductionist challenges: (1) it remains an open question how processes at the biological level are causally dependent on processes at the chemical or physical level as described in these approaches; (2) and even if full causal reduction in the sense of causal dependence could be shown, that leaves unanswered the question to what extent biological *explanations* (or methodologies, concepts, and the like) can or should be reduced to the chemical or physical explanations (or methodologies, concepts, and the like) on offer here. The case of strong reductionist accounts of evolution thus raises general questions about the possible reduction of higher-level theories and fields to more fundamental theories and fields with which philosophers of science have long been concerned. Applying relevant results from philosophy of science to this case can thus be expected to provide more clarity on the feasibility of the DKS approach, formal reductionism, and similar approaches. This work remains to be carried out.

#### 2.3.2. Weak Reducing Accounts

The hypercycle account of Eigen and Schuster [45,46] was one of the first attempts to provide a formal underpinning of evolution at the molecular level—in this case one resting on reaction kinetics. Their theory describes Darwinian behavior at the molecular level in terms of metabolism, self-reproduction, and mutability, which can be understood both conceptually and quantitatively through reaction schemata known as hypercycles. These hypercycles consist of catalytic molecules forming a circular network, in which each type can catalyze both its own formation and the formation of the next type in the network. Crucially, Eigen and Schuster do not argue that evolution at the biological level can be reduced to molecular evolution understood as hypercycles, but rather, they see molecular evolution as one way in which evolutionary processes can occur and leave open the extent to which evolution at higher levels of organization instantiates the same process. In an early publication in which the idea was first introduced, for instance, Eigen specified that the theory explains “the general principle of selection and evolution at the molecular level, based on a stability criterion of the (non-linear) thermodynamic theory of steady states”, and that it may explain “how to construct simple molecular models representing possible precursors of “living” cells” [51]. In this sense, Eigen and Schuster’s approach is merely weakly reductionistic, as it does not aim to reduce all instances of evolution to the same fundamental physical process, but only claims that a fundamental physical process (the hypercycle) lies at the start of the evolution of life on Earth and that the occurrence of evolution was inevitable once hypercycles occurred.

Eigen and Schuster’s account has been hugely influential in origins of life research, both for its emphasis on reaction kinetics, as well as its mathematical approach. Kauffman’s account of complex self-organization [47] can be seen in this tradition, although Kauffman’s account goes further still in its formal character. Kauffman posits his account as one standing alongside Darwinian evolution, explaining the structure of living matter as arising not merely from adaptive evolution, but from self-organizing principles of complex systems. Kauffman provides an abstract model, the NK model, which describes the dynamic behavior of large numbers of interactions, *K*, between large numbers of components, *N*, mapped onto a fitness landscape. By varying the relevant parameters, the behavior of a given system can be described. This approach is purely formal, in the sense that it is not dependent on what type of matter is instantiated in the NK model. It should be noted that Kauffman is explicitly anti-reductionist when it comes to the subject matter of biology as a field of investigation: according to Kauffman, biology cannot be reduced to chemistry, physics, or, for that matter, any single set of principles. Nevertheless, Kauffman’s account does encompass a weak reductionistic element in its aim to cover part of the subject matter of evolutionary theory (the origins of organismal forms and biological structures more generally) by an account of self-organization. Whereas orthodox Darwinian approaches see biological structures as due to random variation and selection, Kauffman sees them as primarily due to self-organization, with a lesser role for variation and selection. A (considerable) part of the explanatory scope of Darwinian evolution is thus reduced to self-organization, leaving other parts intact as proper explananda of evolutionary theory.

Both Eigen and Schuster’s account and Kauffman’s account suffer from a variety of issues. These relate to the molecular assumptions of both accounts, as well as their mathematical underpinnings. These issues have been discussed in Maynard-Smith and Szathmary [36] and include the question of substrate neutrality, the question of what constitutes the proper unit of selection in these accounts, and the way in which either account can give a plausible chemical basis for the origin of life. 

## 3. The Origins of Life and Its Early Development

### 3.1. General Considerations

In view of the diverse approaches and lines of research in origins of life research and prebiotic chemistry, a kaleidoscope of different theories of the origins of life in relation to molecular evolution could here be discussed [52]. The two most dominant of these are the *RNA world* theory [53,54], and the *metabolism first* theory [55]. These fundamental theories and hypotheses seek to delineate the road to the first early forms of life, i.e., to simple protocells that existed before the appearance of LUCA [56]. The RNA world theory suggests that the biomolecules that made evolution possible were RNA molecules, or very similar molecules, which were capable of carrying both catalytic and heritable information. Due to the structural complexity of nucleic acids, a rich prebiotic chemistry must have developed first, and protocells with multilayered metabolic networks only appeared on the scene much later. The default hypothesis to explain how the universal genetic code originated in such a diverse prebiotic chemistry is that it is a “frozen accident”, but other explanations are available [57]. The second hypothesis states that metabolic reaction networks preceded genetic information carriers. These reactions were embedded in a network of self-sustaining cycles that steadily grew in complexity. Occasionally, these reactions split into two independent routes, so that evolution of new compounds or more efficient pathways could have developed from these mixtures of reactions, so that fairly early in evolution, some type of protocells could have formed. For further discussion of our perspective on the “frozen accident” hypothesis and the possible impact of recent prebiotic amino acids, we point the reader to [58].

Here, we do not focus on the RNA- or metabolism-first theories, especially since in our view these should not be discussed as either/or options. Rather, we explore the significance of evolutionary theory for origins of life research through a brief discussion of two stages in the origins and early development of life. First, in Section 3.2, we look at the concept of (multistep) catalysis. Although the concept of catalysis is primarily a chemical one, it is de facto a fundamental element of all (bio)metabolic processes, including self-replicating systems. Catalysis is the engine that kinetically facilitates and promotes reactions and reaction networks. Can catalysis serve as a showcase for how evolutionary theory is applicable to the emergence of prebiotic and early biotic networks? And, if so, are the ideas on evolution that are discussed in the previous section specific enough to reveal the driving forces that transformed a prebiotic world into the first life forms? Second, in Section 3.3, we look at the early, potential evolution of (proto)metabolic networks, also in a protocellular context. There, we again ask how evolutionary theory could be applicable to this phase of the early development of life.

### 3.2. The Role of Catalysis 

Metabolic pathways and cycles, and ultimately metabolic networks, are characterized by many individual reactions in which the product of one reaction serves as a starting material for the next reaction. The effectiveness of such multistep processes strongly depends on the kinetics of the individual steps being adapted to each other. Although not every single reaction in such networks needs to be promoted by a catalyst, catalysts (or in biological systems, enzymes) are nevertheless crucially important to kinetically orchestrate such pathways, cycles, and networks [59,60]. Compared to the landmark experiments of Urey and Miller [61], current research in prebiotic chemistry has developed in at least the following two ways: (1) we search for longer pathways, more extended protometabolic cycles, or networks mimicking biological systems, and (2) metals as catalysts (such as iron or nickel) play an important role [62,63].

Interestingly, arguments for the RNA-first hypothesis are also linked to the paramount importance of catalysis, as RNA in the form of ribozymes also had to act as catalysts to chemically allow metabolic diversity at that time. In addition, early forms of coenzymes and cofactors such as simplified derivatives of pyridoxal phosphate (PLP) or *N*-alkylated nicotinamides (NAD(H)), basically a distinct form of organocatalysts, as well as iron sulfur clusters, must be included in the evolution of catalysts in the transition from the abiotic to the biotic world [64]. Within the concept of the RNA world theory, these may also have been a part of RNA either through covalent bonds or through weaker interactions, such as those found today in riboswitches [65,66]. 

The element iron is an exemplary candidate for the molecular evolution of catalysts that begins in the prebiotic world and leads to early forms of biocatalysts that are involved in key biochemical processes that still exist today, and here, we use iron and iron sulfur clusters as a molecular showcase of potential evolution [67]. Purely inorganic [4Fe4S] species, as present in the thiospinel lattice of greigite, may have played a vital role in protometabolism before LUCA [56]. These clusters became a part of early forms of life as electron transfer cofactors [68,69,70]. These [FeS] clusters developed into catalysts that were able to bind and process molecular hydrogen in the early stages of life [71], allowing for nitrogen fixation [72,73] and the binding and reductive processing of carbon dioxide in the ancient Wood–Ljungdahl pathway [74,75]. A key development included the replacement of individual iron atoms with other metals such as molybdenum, vanadium and, above all, nickel, as found in [FeNi]-hydrogenases. 

In the biological world, the duplication and divergence of genes are tools to create new catalysts/enzymes. What could have enforced the development of chemical catalysis and the appearance of new or more specific catalysts in the prebiotic world? The chemical and geological environments and fluctuating physicochemical conditions such as temperature, pressure, pH, and UV radiation could have been the driving forces for the emergence of new catalysts, new reactions, and thus, new organic molecules. Several geological sites and conditions have been considered for the appearance of ever more complex organic molecules and catalysts in a prebiotic world. These are deep-sea vents (black and white smokers), but also hydrothermal fields which, unlike deep-sea vents, are exposed to photochemical conditions and can undergo wet and dry cycles. Other sites to be considered are tectonic faults and cold geysers, as well as the fluctuating conditions on the sea coasts, which were much more pronounced due to the greater gravity fluctuations that were caused by the moon being closer to Earth at the time [76].

It is tempting to understand the development of chemical catalysts along selectionist lines. Provided that prebiotic catalysts could influence their environment in such a way as to promote their own formation, these catalysts could be described as being selected for, or as having adapted to their environment. However, is such a scenario a consequence of chemical necessity [77] or of chemical evolution akin to biological evolution? As discussed in Section 2.1, selection and adaptation are parts of ENS, but so are notions such as fitness and heritability. If we consider heritability, for example, it is unclear what it could mean for a parental catalyst to pass on heritable traits to its catalytic offspring. This seems to require some notion of compartmentalization designating a clear unit of selection, as well as a genetic component signifying the functional catalyst [36].

### 3.3. Protometabolic Networks, Genetics, and Protocells 

Recently, systems chemistry and computer-predicted prebiotic synthesis have been introduced into the field of prebiotic chemistry, going beyond the study of individual reactions [78,79,80,81,82]. This trend not only considers prebiotic pathways to selected molecules and oligomers, but rather seeks cooperative interactions and networks between different classes of molecules. These metabolic pathways are relics of a self-organized reaction network that developed spontaneously before enzymes existed [81,82]. In this scenario, the current enzymes are replaced by naturally occurring minerals or metal ions. For example, the reductive tricarboxylate cycle [83], the Wood–Ljungdahl pathway [74,75,84,85], glycolysis [86], and the glyoxylate cycle [87] have been proposed as such biological metabolic cycles or networks. Some aspects, particularly primordial metabolic cycles, have triggered controversial debates [67,88,89]. Orgel pointed out that abiotic reactions take place with low yields, i.e., the more reaction steps that proceed linearly one after another, the more catastrophically the total yield decreases. This is a particularly dramatic problem for metabolic cycles, since the substrate concentration for the first step depends entirely on the yield of the last step, a fatal situation for primordial metabolic cycles.

But why and in what way could an early metabolism evolve (one that would include the development of suitable catalysts)? And how did metabolic networks become more complex? Can we apply the evolutionary principles outlined in Section 2 to understand what enabled so-called chemical evolution and its connection to the emergence of protobiological metabolic pathways and networks? Before going into this question, we first sketch five hypotheses for metabolic evolution that combine prebiotic elements from the previous section with genetic and protocellular considerations. 

#### 3.3.1. The Retrograde Hypothesis

**Figure 1:**
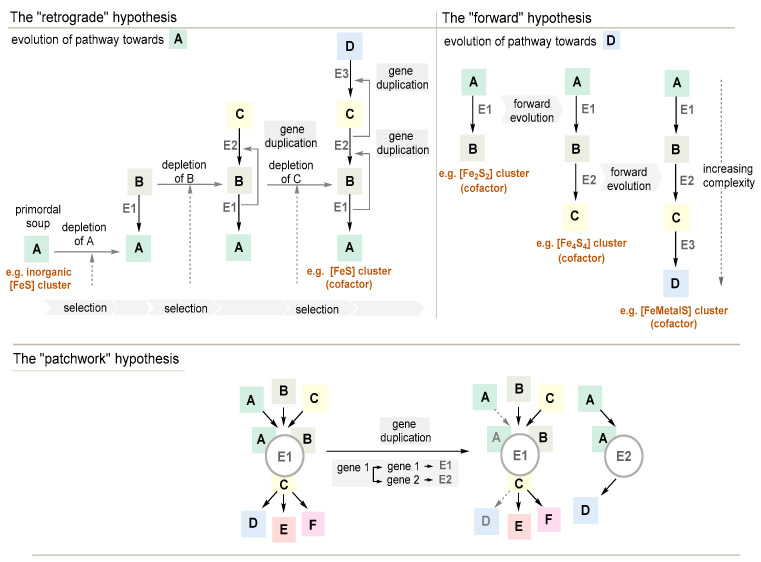
Three hypotheses of metabolic evolution, and where appropriate, a reference to iron sulfur cluster development is given. Top left: the retrograde hypothesis of metabolic evolution (A–D = molecules of a biosynthetic pathway, with A being the end product; E1–E3 = enzymes). Top right: Granick’s forward hypothesis of metabolic evolution (A–D = small molecules that are of a biosynthetic pathway, with A being the end product; E1–E3 = enzymes). Bottom: the patchwork hypothesis of metabolic evolution (A–C = precursor molecules; D–F = products of an enzymatic transformation; E1 represents the ancestral catalyst which endowed substrate promiscuity that was able to catalyze three different, but similar reactions; E2 represents the next generation of enzymes, in which the amino acid sequence has diverged slightly and substrate specificity and catalytic activity have been increased).

Horowitz suggested a theory of evolution with reference to biomolecular networks [90]. The “retrograde” theory of evolution states that the first living species was a completely heterotrophic organism that reproduced at the expense of prebiotically formed organic molecules. This refers to molecule A, which is essential for survival (Figure 1, top left). In this context, amino acids, a few prebiotically formed molecules with properties of coenzymes and cofactors such as [FeS] cluster can be listed. This protoorganism will consume environmental reserves of A. Thus, A will be depleted to a point where growth is limited, so that any organism that evolves an enzyme or catalytic system that is capable of synthesizing a molecule A from precursors B would have a selective advantage. It would rapidly grow and dominate its environment. As a consequence, metabolic pathways must have arisen through successive gene duplications. This selection process could be repeated for subsequent generations until the biosynthetic pathway known today was established. Horowitz’s theory provides an additional aspect in that further evolution is likely to be based on a random combination of genes. Here, simultaneous unavailability of two intermediates (e.g., B and C) could result in a symbiotic association between two mutants. One would be capable of synthesizing B, and the other one would be involved in synthesizing C from other precursors in the environment. Consequently, the evolution of short reaction chains would occur and dominate using molecules whose synthesis has been previously acquired. It is important to note that this theory incorporates the idea of parasitism and symbiosis as driving forces of evolution [91]. In the context of this account, the retrograde hypothesis is important as it provides an evolutionary link between prebiotic chemistry and the development of early metabolic pathways. 

However, elements of this theory have been critically commented upon. As it postulates that the evolution of metabolic pathways proceeded in the reverse direction, it would require particular environmental conditions. Prebiotically generated potential precursor molecules such as [FeS] clusters accumulated, but they depleted over time as protometabolic networks and early forms of life developed, thereby stopping the evolutionary process. Furthermore, in many cases, the origin of anabolic metabolic pathways cannot be inferred from their backward evolution. This is due to the fact that such pathways proceed via unstable intermediates, and it would be difficult to explain their accumulation in both prebiotic and contemporary environments. Furthermore, if catalysts that promote consecutive steps in a given metabolic pathway have evolved from a series of gene duplications, it is only reasonable to assume that they must have structural similarities. In reality, the list of known examples of homologous adjacent enzymes in a metabolic route is rather small.

#### 3.3.2. The Forward Hypothesis

A less well-known hypothesis by Granick negates the importance of prebiotic compounds in biological evolution [92]. It states that the emergence of a biosynthetic pathway towards the end product(s) D is driven by forward evolution—basically from simple precursors A via B and C to complex molecules D (Figure 1, top right). Enzymes E1 or E2, catalyzing earlier steps in a metabolic pathway, are older than E3 and E4 that operate later. Therefore, each intermediate metabolite in a biosynthetic pathway must be useful to the organism or in its evolution. This is due to the fact that the simultaneous evolution of several genes in a sequence is rather unlikely. Granick pointed out that this hypothesis may be questionable for complex, linear biosynthetic pathways, e.g., those leading to purines and the branched-chain amino acids, where the intermediates have no obvious benefit to the organism [93]. The forward hypothesis may very well have operated in the evolution of [FeS] cluster architectures, where the dimerization and recruitment of other metals than iron would represent evolutionary progress [94].

#### 3.3.3. The Patchwork Hypothesis

Thirdly, the patchwork hypothesis also places gene duplication at the center of the evolutionary enforcement of increasingly complex metabolic pathways and networks [95,96]. Here, metabolic pathways emerged through the recruitment of primitive promiscuous enzymes that could react with a broad range of chemically related substrates (Figure 1, bottom). In terms of turnover, their catalytic capabilities would still have been low. However, they guaranteed the functioning of a primitive metabolism in primordial cells with genomes that were still small.

It was noted that the patchwork hypothesis provides good arguments for understanding the evolutionary development of biosynthetic routes towards proteinogenic amino acids. Genome sequence analysis showed that a significant percentage of metabolic genes may have been the result of paralogous duplications. These cover enzymes that catalyze various reactions in the biosynthesis of threonine, tryptophan, isoleucine, and methionine, of which three belong to the aspartate family [74,97]. The patchwork hypothesis is mainly valid for understanding the evolution of metabolic pathways. It fails to explain the transition from prebiotic chemistry to early forms of protein biosynthesis and the emergence of enzymes. 

#### 3.3.4. The Mixed Origin of Metabolic Pathways

The patchwork hypothesis also strongly focuses on enzyme evolution, trying to include prebiotic chemistry and the appearance of the first enzymes [98,99]. For this, it was assumed that the repertoire of available building blocks consisted of stable prebiotically generated molecules, as well as molecules derived from existing metabolic pathways in cells for which stability was not a mandatory requirement. The expansion of the metabolic repertoire should have occurred by gene duplication and divergence and should have produced non-specific protoproteinaceous catalysts, where it was supposed that these protoproteins were formed by non-enzymatic reactions [100,101]. 

In some ways, this mixed-origin approach is exemplified for iron sulfide clusters discussed in Section 3.1. Dimeric and tetrameric iron sulfur clusters are known to form under abiotic conditions, and they exert redox properties without being embedded in a protein template. Only when the first proteins emerged—how this might have happened is not the issue here—did the [FeS] clusters transform into cofactors that were embedded in early enzymes. These could perform electron transfer processes that the protein alone could not accomplish. From there, adaptive changes led to the formation of active sites within the protein that were able to accommodate more complex clusters or additionally allowed to recruit other metals. From the abiotic supply of these first clusters, evolution led to enzyme-catalyzed routes towards [FeS] and [FeMetalS] clusters, so that the biotic world gained complete control over their syntheses [102].

#### 3.3.5. The Shell Hypothesis and a Proposal for a Modification 

**Figure 2:**
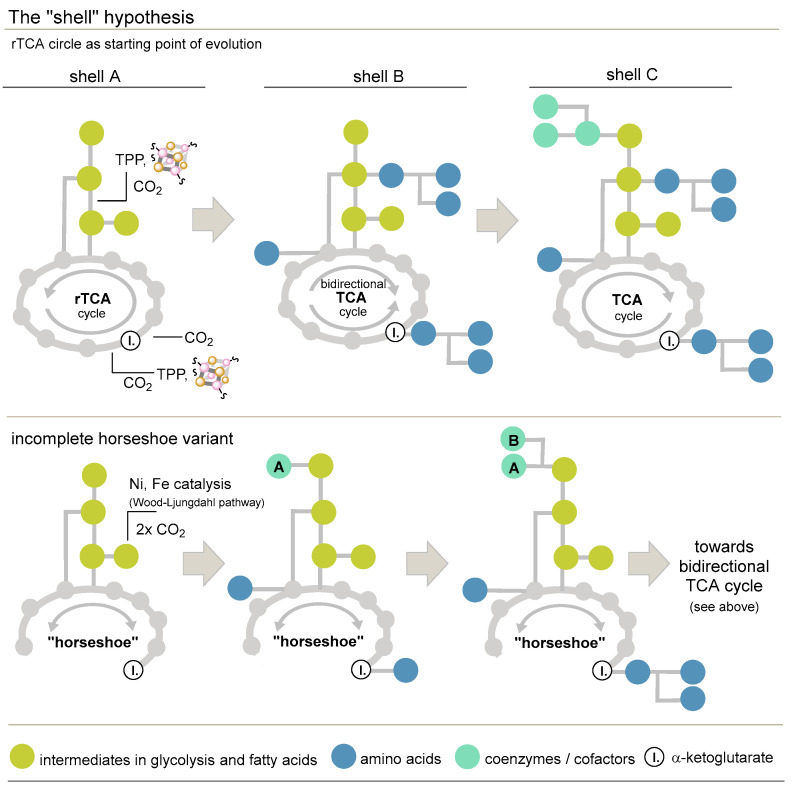
Top: Morowitz “shell” hypothesis that is based on the rTCA cycle and location, where the coenzyme thiamine pyrophosphate (TPP) is operative. Bottom: Transfer onto the incomplete “horseshoe” cycle. Coenzymes labeled with numbers A and B supposedly appeared early in evolution, e.g., nicotinamide and pyridoxal phosphate.

Finally, the shell hypothesis reflects an approach that focuses specifically on the reversed tricarboxylic acid (rTCA) cycle (Figure 2). Besides the non-cyclic Wood–Ljungdahl pathway, this cycle is considered the second candidate for primordial C1 fixation. The shell hypothesis states that the rTCA cycle represents an “energy-amphiphilic” core that reveals itself as a starting point for extended metabolism through the formation of new molecules (Figure 2, top) [103]. This hypothesis may be regarded as a kind of theoretical incubator for today’s efforts of prebiotic chemists [77]. It assumes that the prebiotic chemical processes are “imprinted” on modern metabolism as relics [78,99]. The first shell, A, that was fed from the rTCA cycle supposedly included glycolysis and fatty acid biosynthesis [72,73,74,100]. Next, the introduction of nitrogen linked to amino acids occurred, which formed shell B, and eventually, sulfur was introduced in shell C. As a consequence, purines, pyrimidines, and many other cofactors or coenzymes formed as relative evolutionary latecomers. In the further course of molecular evolution, the rTCA changed via a transition phase to a bidirectional cycle, and finally, the TCA cycle prevailed. At this stage, minerals, metal ions and proto-coenzymes were replaced by current enzymes.

So far, synthetic efforts have failed to prove that the full rTCA cycle can be mimicked experimentally under plausible prebiotic conditions [77], and even if this were to succeed in the future, Orgel’s objections remain unchanged, which state that primordial cycles are in a fatal situation, as they are faced with a constant reduction of substrate concentration for the first step, because they depend entirely on the yield of the last step [83]. In addition, a theoretical study on the close evolutionary relationship between amino acid metabolism and the availability of selected coenzymes, in particular thiamine pyrophosphate (TPP), a biological latecomer, casts doubt on the primordial role of the rTCA cycle, particularly because it also depends on the availability of TPP (2-oxoglutarate:ferredoxin oxidoreductase and *pyruvate: *ferredoxin oxidoreductase** (PFOR)) and Fe_4_S_4_ clusters [54,101]. A way out of these various dilemmas would be to consider a non-cyclic, horseshoe version of the TCA cycle that has prevailed in *Elusimicrobium minutum* until today [102] (Figure 2, bottom). It contains a reductive (via oxaloacetate) and an oxidative branch (via citrate), while the Wood–Ljungdahl pathway, the only non-cyclic C1 fixation pathway known, would provide acetyl-CoA. The sole focus on early metabolic cycles would be circumvented, and the coevolution of coenzymes and cofactors would be taken into account in this hypothesis [54,103].

The status of genetics in the accounts presented above warrants special attention. While the initial stages of catalytic development are still understood in prebiotic terms, such as the primordial soup phase of the retrograde hypothesis (Figure 1, top right), the later stages are understood along genetic lines: complex organocatalysts or protoproteins develop through changes at the genetic level, such as gene duplications. While this brings us closer to extant biology, and so to ENS, it does beg the question as to what changed in between the non-genetic primordial soup, and the genetic selection of organocatalysts and protoproteins. Moreover, without compartmentalization, it remains unclear what could be the unit of selection in these systems. 

Comparing the catalytic story told here, is it possible to relate some of the hypotheses on the formation of metabolic pathways and networks to the evolutionary theories presented in Section 2? For a strongly reductive approach such as the DKS account of Pross and the formal account of Vanchurin and coauthors, it is challenging to provide a clear connection to the prebiotic metabolic hypotheses that are explored here. As we have stated above, this is partly due to a lack of critical examination of these accounts, but there are other reasons. In the case of the DKS account, it is unclear how dynamic kinetic stability would direct the development of complex metabolic networks, and how we might use it to distinguish between the various hypotheses that are presented here. In the case of the formal reduction account, it remains unclear what it means precisely for the metabolic hypotheses discussed here to be learning systems, in the relevant sense. 

The weakly reductionist account of Eigen and Schuster, but also that of Kauffman, can be more easily connected to the topic of metabolic development. Indeed, autocatalysis, a catalytic process in which the product also serves as a catalyst for its own formation, such as the well-known formose reaction, can be regarded to be a reasonable link. The extended version of autocatalysis would be an autocatalytic set in which several collectively autocatalytic reactions operate across different reactions which are embedded in the hypercycle theory. In principle, every catalytic step in the retrograde, the forward, and the patchwork hypotheses could be autocatalytic or be part of hypercycles in Eigen’s sense. However, Eigen´s approach was discussed for molecules that depend on and cooperate with each other through feedback, while metabolic pathways, networks, or cycles resembling extant metabolic networks were not a focus. Furthermore, one has to acknowledge that only very few autocatalytic reactions are known so far, of which the formose reaction is likely the most relevant in the present context, even if autocatalytic sets of reactions have been identified within extant prokaryotic metabolism.

The number of building blocks that give us a larger picture of the transition from an abiotic environment to the biotic world of LUCA is increasing; however, the picture fed by a variety of theories of evolution and hypotheses on the formation of metabolic pathways and networks itself remains very diffuse. In conclusion, it must be stated that a comprehensive theory of evolution and, thus, a continuous story starting from prebiotic chemistry and moving via speculative primordial forms of life to extant life cannot yet be told.

## 4. The Use of Evolutionary Concepts in Origins of Life Research

As shown in the preceding sections, terminology from evolutionary biology is being used increasingly in origins of life research, as well as in other fields concerning aspects of life, such as synthetic biology [11,104,105]. This is not entirely surprising, given that evolution is often seen as a hallmark of life [106]. Yet a number of important, critical questions about the use of evolutionary terminology in origins of life research present themselves: What evolutionary terminology is in fact being used? How is this terminology used? How, if at all, is this use of evolutionary terminology justified? And, more generally, in what ways could it be justified? Our aim here is not to give a complete overview or exhaustive analysis of the use of evolutionary language in origins of life research, as this would be a project in and of itself that we hope to undertake at a later point. Rather, we aim to bring out several important distinctions and to provide several important examples of how evolutionary terminology is currently being used in the origins field. 

We begin with the more general consideration: how could the use of evolutionary terminology be justified? In other words, what potential roles could evolutionary terminology play in (some parts of) origins of life research? There are at least four ways to think of the use of evolutionary terminology, running from strongest to weakest in terms of justification: ontological, epistemological, heuristic, and metaphorical. Starting with ontological use, we might say that, ideally, the use of evolutionary terminology in origins of life research is justified because the kinds of processes that are being described simply *are* evolutionary. If origins of life researchers are ontologically justified in using evolutionary terminology, the explananda of life’s origin are sufficiently similar to those of regular evolutionary biology to merit applying terminology from the latter to the former (cf. Section 2.1, [7,28,29]). In the case of epistemic use, evolutionary terminology is justified because origins of life researchers come up with genuine evolutionary explanations, those that generate genuine knowledge and understanding of an evolutionary sort. Thus, there is sufficient similarity between the explanantia in the origins of life field and those in evolutionary biology to warrant the use of evolutionary terminology. Notice that it is unclear to what extent evolutionary explanations in origins of life research can be genuine without there being sufficient similarity between the origins and early development of life and evolutionary biology on an ontological level (cf. [6]). It seems that a prerequisite for the formulation of evolutionary explanations for phenomena that are under consideration is that these phenomena themselves should be evolutionary phenomena. Ontological and epistemological justifications for using evolutionary terminology thus seem intimately connected.

If both the ontological and epistemological uses of evolutionary terminology cannot be justified, then another option might be heuristic use. On a heuristic understanding of the use of evolutionary terminology in origins of life research, the added value is not explanatory in an epistemological or ontological sense, yet the use of evolutionary terminology is somehow conducive to understanding within the origins of life field. The assumption here is that the origin and early development of life are to some extent evolution-like processes, that can be described in evolution-like terms, even if ultimately, they are not properly evolutionary processes. This use gives rise to the question to what minimal extent a process must be evolution-like for it to be meaningfully described in evolution-like terms—in other words, under what conditions a heuristic use of evolutionary terminology can be successful. This evolutionary terminology would certainly be applicable to chemical evolution, as discussed in Section 3.2. 

Finally, we may say that the use of evolutionary terminology in the origins field is merely metaphorical. It is a way of speaking about the origin and early development of life, but it carries no ontological, epistemological, or even heuristic weight. The question then becomes what use evolutionary terminology would be in origins of life research if it does not even serve heuristic purposes. Here, we can distinguish between benign and detrimental use. If evolutionary terminology is benignly metaphorical, its use lies in the area of communication but has little to no consequences for progress or understanding in the origins field. In such cases, the question of justification seems moot, as evolutionary terminology does not carry any import but is merely a tool for communication. If it is detrimentally metaphorical, i.e., it hampers progress in the field or the scientific understanding of life as a natural phenomenon by misleadingly suggesting that the phenomena under study are evolutionary in ways that they are not, then the use of evolutionary terminology should actively be opposed.

It might be the case that evolutionary terminology applies to some parts of the origins and early development of life, but not others. One reason for this is that justification of the use of evolutionary terminology is also strongly dependent on how the origin of life and its early development are conceptualized. This is something that became clear in our discussion of catalysis in Section 3. Evolutionary terminology might straightforwardly apply to some Szostak-cum-Szathmary protocellular part of the origins of life [36,107], with a clear genetic component and compartmentalization, but not to a prebiotic, metabolic network phase (cf. [108]). On this distinction, the use of evolutionary language in protocellular research might even be ontologically justifiable, but the same use of language in prebiotic, metabolic network research might not even be heuristically justifiable.

Looking at recent empirical work on the origins of life—here understood to include “wet-lab” experimental work as well as computational approaches—several things stand out: (i) If evolutionary terminology is used in the origins of life literature, then some terms from ENS are used frequently, while others are rarely used. (ii) There is a marked difference between how explicitly the use of evolutionary terminology is justified in experimental work, where evolutionary terms are used often but justification is rare, compared to reviews and perspectives, where both use and justification are more prevalent. (iii) When comparing experimental work to computation approaches, the use of evolutionary terminology is less explicit in the former than in the latter.

If evolutionary terminology is used in experimental origins of life research, the two most frequently occurring terms are “Darwinian” and “evolution” (or “evolutionary”), often but not always in combination. These terms can often be found in the title of the work [10,109,110,111,112,113,114,115], but they can also appear exclusively as part of the main text [116,117,118,119]. In either case, these two terms are usually part of an explanation of why the work is an example of [109], a step on the way towards [117], or somehow relevant to [111] an evolutionary understanding of the origin of life. Sometimes these terms appear on their own, without invoking other evolutionary terminology, but more often than not, experimental work mentioning “Darwinian evolution” also involves terminology such as “heredity” [114,120] or “selection” [115,116]. Interestingly, terms that seem to be less common in experimental work are central terms in evolutionary biology such as “variation” [120], “mutation” [119], “competition” [117], and explicitly, “natural selection” [120]. 

However frequent or infrequent an evolutionary term may be used in experimental work within the origins field, the extent to which this use of terminology is justified varies greatly (compare, for instance [109,110,112,118]). A good example of this are two experimental papers about a very similar system, one by Ichihashi and coauthors [109] and one by Matsumura and coauthors [118]. 

Beginning with the more recent of the two: Ichihashi and coauthors combined the Qβ RNA replicase system with a purified translation system and showed that genomic RNA can outcompete parasitic RNA for the replicase when RNA replication takes place in compartments going through manually induced cycles of division and fusion. They argue that their replicating system is developed through Darwinian evolution, and although they do not provide a fully developed ENS-type account, some effort is made to explain why they believe their system to be evolving. Specifically, they take error-prone replication to stand at the core of evolution at the molecular level, and they argue that their system behaves similarly to bacterial species, as the increase in the rate of replication (the fitness increase) plateaued, while the rate of mutation remained more or less constant. Regardless of whether this stands as a justification for their use of evolutionary terminology, it is clear that some effort was made to justify this use. 

Matsumura and coauthors describe a very similar system, which predates that of Ichihashi and coauthors, and, contrary to Ichihashi and coauthors, they do not go as far as saying that their system is undergoing Darwinian evolution. Nevertheless, their replicating system is described in evolutionary terms and it is supposed to be a plausible example allowing for “the evolution of molecular complexity before the first protocells” [54] (p. 1293), with the rest of the results being phrased in terms of selection, parasites, and extinction. All-in-all, little of this language is explicitly justified. The point here is not to adjudicate which of these uses of evolutionary terminology is justified, but to bring out how differently the question of justification is treated in experimental origins of life research. Interestingly, this lack of justification is usually more pronounced in experimental work than in reviews and perspectives on evolution and the origins of life [108,121,122,123], although here too, discussion can be sparce to minimal [124,125,126].

Notwithstanding the variety of evolutionary terminology that is used, and the variety in how extensively this is justified, there are examples of a seemingly more coherent and durable use of evolutionary terminology in experimental origins of life research. Perhaps the clearest example of this, and one that certainly deserves special consideration, is the work of Sijbren Otto [9,119,127,128,129,130,131,132,133,134,135,136]. Over the past decade, he has developed a surprisingly versatile chemical system, which he claims shows many of the hallmarks of an evolutionary system, or at least has the potential to become an evolutionary system. The system is built around a peptide consisting of alternating hydrophobic and hydrophilic amino acids, connected to a pendant thiol group. Through the pendant thiol group, these peptides can form differently sized rings called macrocycles. Macrocycles of the same size can form into fibers due to β-sheet formation along the axis of the fiber based on the peptide sequence [127]. Surprisingly, for a given macrocycle size and depending on the precise conditions, the fibers that are produced from these macrocycles can catalyze the formation of the macrocycles that are built into the fibers, meaning that these fibers can be thought of as replicators [128]. Otto and coauthors discuss the possibility of Darwinian evolution using the macrocycle system: “The newly developed family of replicators opens up exciting possibilities for achieving Darwinian evolution in a fully synthetic system of self-replicators.” (idem, p. 18414). While they are aware that their system is not a plausible candidate for the origin of life, they do claim that it could aid us in understanding evolution at the chemical level [135]. In subsequent research, Otto and coworkers study replicator competition in relation to fitness [129], replicators as species occupying a certain “food” niche [119], replicators as parasites and predators [131], replicators as constituting a protometabolism together with a cofactor [134], and replicators creating an eco-evolutionary dynamic [9]. Throughout this work, claims about the evolutionary status of the system vary in strength, and Otto is certainly aware that open-ended evolution remains an outstanding challenge [136]. While Otto’s use of evolutionary terminology is extensive, it remains an open question to what extent this use can be justified.

Another interesting difference in the use of evolutionary terminology in the origins field is that between the use and justification of evolutionary terminology in experimental work and computational work. Computational approaches to studying the origin and early development of life are often focused on the general conditions for self-replication and the requirements for self-replication to be evolutionary [137,138,139,140,141,142,143,144]. Several papers by Vasas and coauthors represent an especially clear example of explicit justification of the use of evolutionary terminology [136,137,138,139]. Their work presents one of the few examples where it is explicitly stated that care should be taken in applying evolutionary terms beyond evolutionary biology [140], something that they have put to effect in arguing that autocatalytic networks can evolve under the right conditions [142]. Most interestingly, however, is their discussion of the requirements for evolution in [141], where they specify that for a chemical system to evolve requires not just selection, but also open-ended evolution, which is itself possible on the condition of “a very rich combinational generative mechanism… [with] unlimited heredity; namely, that the number of possible heritable types should more than astronomically exceed the number of individuals in the population… [and] an inexhaustible fitness landscape. By this we mean that as evolution proceeds, there should be newer and newer possibilities for empty niches.” (idem, p. 37). Given that these are plausible conditions, this also explains why Otto’s macrocycle system is far removed from an open-ended system, as it fails to be a sufficiently rich combinatorial generative system, and it is a clear example of limited rather than unlimited heredity.

One plausible explanation for why evolutionary terminology is more explicitly justified in computational approaches than in experimental approaches lies with the different nature of these two types of approaches. In the case of experimental work, its relevance does not rest entirely (and perhaps not at all) with the results being appropriately evolutionary, for even if they are not, experimental results may still be interesting from a chemical, biological, or physical perspective. This does not apply to results of computational approaches in quite the same way, as the value of computational results is heavily dependent on the appropriateness of the assumptions of the computational model. If these assumptions are meant to be evolutionary in some important sense, this has to be adequately specified or else any conclusions drawn are of little value.

While this explanation may be adequate to explain the difference between the use and justification of evolutionary terminology in experimental versus computation work, it does not explain why there are such large differences between the use of and justification of evolutionary terminology within experimental origins of life research. Looking at experimental work with little to no justification provided for the evolutionary terminology that is being employed, there seems to be a tacit assumption on part of the authors that it is “clear enough” what constitutes evolution. However, as we have seen in Section 2, and as has been made clear by the work of Lewontin [20] and Godfrey-Smith [21,22], even if evolution is understood as ENS, its precise conditions of application are not clear. Of course, even the assumption that evolutionary theory can be reduced to some form of ENS has been extensively questioned [3,4,5]. If, as we have argued, the standard for the application of evolutionary theory should be set first and foremost by evolutionary biology, then the question is how large a part of evolutionary theory at the biological level we may forget when applying evolution at the origins of life.

## 5. Concluding Remarks

In this review, we have attempted to bring together work on the application of evolutionary theory outside biology from a number of different disciplines, including philosophy of science, evolutionary biology, and origins of life research. Our aim was to bring out several interesting questions and open new avenues of research. We believe that several important conclusions can be drawn. First, as seen in Section 2, it is not straightforward to apply evolutionary theory beyond biology, whether evolutionary theory is generalized or reduced. Both of these approaches face a number of difficulties in specifying a version of evolutionary theory which remains similar enough to evolutionary biology to deserve the moniker. Second, as seen in Section 3, when looking at a concrete question within the origins of life field, it is not straightforward to apply the various extended accounts of evolutionary theory that are on offer. Third, as seen in Section 4, the extent to which the use of evolutionary terminology is justified differs widely between different experimental approaches, as well as between experimental and computation work and between reports of experimental work and reviews and perspectives.

In drawing these conclusions, we hope to have introduced a helpful way of classifying various extended accounts of evolutionary theory in terms of their generalizing or reducing character. Similarly, we hope to have provided some clarity on the various ways in which the terminology from evolutionary theory can be of use in origins of life research, whether ontologically, epistemologically, heuristically, or metaphorically. All in all, this review should contribute to a clearer and deeper understanding of the relevance of combining philosophical, theoretical, and empirical considerations in studying evolution at the origins of life.

## Data Availability

Not applicable.
